# The Neovolcanic Axis Is a Barrier to Gene Flow among *Aedes aegypti* Populations in Mexico That Differ in Vector Competence for Dengue 2 Virus

**DOI:** 10.1371/journal.pntd.0000468

**Published:** 2009-06-30

**Authors:** Saul Lozano-Fuentes, Ildefonso Fernandez-Salas, Maria de Lourdes Munoz, Julian Garcia-Rejon, Ken E. Olson, Barry J. Beaty, William C. Black

**Affiliations:** 1 Department of Microbiology, Colorado State University, Fort Collins, Colorado, United States of America; 2 Laboratorio de Entomologia Medica, Faculdad de Ciencias Biologicas, Universidad Autonoma de Nuevo Leon, Monterrey, Mexico; 3 Departmento de Genetica y Biologia Molecular, Instituto Politecnico Nacional, Mexico City, Mexico; 4 Centro de Investigaciones Regionales, Universidad Autonoma de Yucatan, Merida, Mexico; Duke University-National University of Singapore, Singapore

## Abstract

**Background:**

*Aedes aegypti* is the main mosquito vector of the four serotypes of dengue virus (DENV). Previous population genetic and vector competence studies have demonstrated substantial genetic structure and major differences in the ability to transmit dengue viruses in *Ae. aegypti* populations in Mexico.

**Methodology/Principal Findings:**

Population genetic studies revealed that the intersection of the Neovolcanic axis (NVA) with the Gulf of Mexico coast in the state of Veracruz acts as a discrete barrier to gene flow among *Ae. aegypti* populations north and south of the NVA. The mosquito populations north and south of the NVA also differed in their vector competence (VC) for dengue serotype 2 virus (DENV2). The average VC rate for *Ae. aegypti* mosquitoes from populations from north of the NVA was 0.55; in contrast the average VC rate for mosquitoes from populations from south of the NVA was 0.20. Most of this variation was attributable to a midgut infection and escape barriers. In *Ae. aegypti* north of the NVA 21.5% failed to develop midgut infections and 30.3% of those with an infected midgut failed to develop a disseminated infection. In contrast, south of the NVA 45.2% failed to develop midgut infections and 62.8% of those with an infected midgut failed to develop a disseminated infection.

**Conclusions:**

Barriers to gene flow in vector populations may also impact the frequency of genes that condition continuous and epidemiologically relevant traits such as vector competence. Further studies are warranted to determine why the NVA is a barrier to gene flow and to determine whether the differences in vector competence seen north and south of the NVA are stable and epidemiologically significant.

## Author Summary

The Neovolcanic axis (NVA) traverses Mexico at the 19^th^ parallel and is considered to be a geographic barrier to many species. We have demonstrated that the intersection of the NVA with the coast in Veracruz state is a barrier to gene flow in *Ae. aegypti*. This was unexpected because the intersection of the NVA with the Pacific Coast is not a barrier to gene flow. Further studies to identify the actual mechanism(s) that is(are) contributing to the lack of gene flow will provide important information on the trafficking potential of *Ae. aegypti*, which will be of great value to *Ae. aegypti* control programs. There are significant differences in vector competence for dengue virus between mosquitoes north and south of the NVA, but the epidemiological significance of these finding remains to be determined. Future studies will determine if, for example, the genes that condition midgut infection and vector competence of *Ae. aegypti* populations provide biomarkers for risk of dengue transmission. Such biomarkers could be of great value to control programs in resource limited environments by allowing targeting of vector control efforts to areas at most risk for epidemic dengue and dengue hemorrhagic fever.

## Introduction

The mosquito *Aedes aegypti* is the main vector of the four serotypes of Dengue virus (DENV1-4). There are 50–100 million DENV infections each year [1],[2] and while most of these are mild or asymptomatic, the numbers of severe infections with shock and hemorrhage have increased dramatically in many parts of the world [3],[4]. *Aedes aegypti* populations exhibit a large amount of genetic variation in their ability to become infected with, propagate, and eventually transmit flaviviruses [5]–[8], including DENV1–4. Vector competence for flaviviruses is thought to be controlled by at least two physiological mechanisms, a midgut infection barrier (MIB) and a midgut escape barrier (MEB) [9],[10] with environmental factors contributing up to 60% of variation [9]. Our genetic studies suggested that infection rates among natural populations of *Ae. aegypti* may be due to segregation of alleles at up to 8 loci [11]–[14].

We previously conducted studies to determine the breeding structure and vector competence of *Ae. aegypti* populations in Mexico [15],[16]. For the population genetic studies, *Ae. aegypti* were collected from throughout the coastal regions of Mexico, and 25 haplotypes of the Nicotinamide Adenine dinucleotide dehydrogenase subunit 4 mitochondrial (ND4) gene were detected by SSCP analysis. These studies revealed that northeastern Mexican *Ae. aegypti* were genetically differentiated from the Yucatan and Pacific Coast mosquitoes. F_ST_ values revealed extensive gene flow along the Pacific Coast, but not in the Yucatan Peninsula and northeastern Mexico. These studies also revealed a barrier to gene flow somewhere along the Gulf of Mexico between Tuxpan and Moloacan/Minatitlan in northern and southern Veracruz State, respectively. *Ae. aegypti* collected for the population genetic studies were also phenotyped for vector competence for DENV2, which revealed considerable variation in vector competence for DENV2 in Mexico [8]. Interestingly, the *Ae. aegypti* collections from southern Veracruz Coastal Plain differed significantly in vector competence; mosquitoes from Merida, Chetumal, and Cancun in the Yucatan were the most vector competent and those from Nuevo Laredo and Houston, the least vector competent [8]. Unfortunately, in both the population genetic and vector competence studies, no sites were sampled in Veracruz state (∼750 km from north to south). This prevented us from identifying the specific barriers to gene flow.

This also prevented us from examining vector competence in Veracruz. This is of special interest because a 1986 serological survey conducted by the Secretaria de Salud of Mexico [17] revealed major differences in dengue seroprevalence rates in cities and towns in Veracruz state. The dengue seroprevalence rate was 58% (29/50) in people from Martinez de la Torre (in northern Veracruz) versus 0% (0/50) in samples from Moloacan (southern Veracruz).

**Figure 1:**
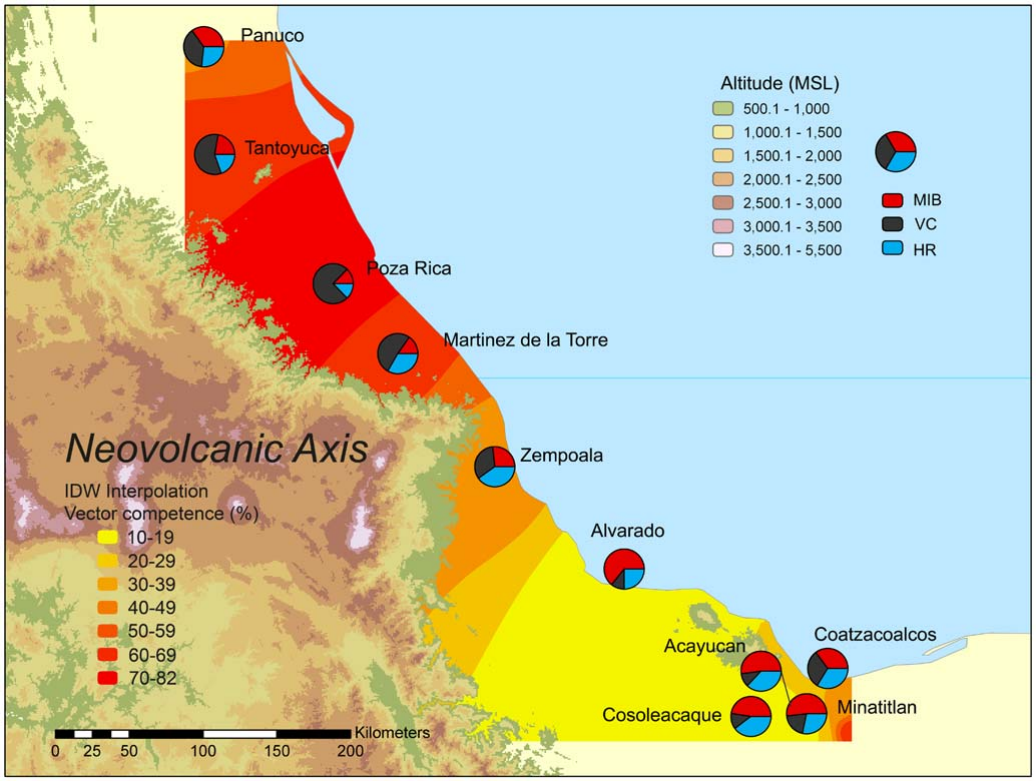
Map of the coastal plain of Veracruz indicating the locations of the 10 *Aedes aegypti* sampling sites relative to the Neovolcanic Axis. Pie charts indicate the proportion of mosquitoes that were vector competent (black), midgut negative (red) and head negative (green). The VC rates were interpolated by Inverse Distance Weighting and geographic areas are colored from yellow to red according to predicted vector competence rates. R^2^ = 0.66 and root mean square error = 9.6.

The present study is therefore an attempt to define more precisely the geographic barrier to gene flow previously observed between the northern [16] and southern Gulf of Mexico Coastal Plain [15] and to characterize more thoroughly the vector competence of mosquitoes separated in southern Veracruz. We obtained 10 *Ae. aegypti* collections between Tuxpan in the north to Minatitlan in the south (Figure 1). Nine of these same 10 sites were resampled in 2004 to test the consistency of our 2003 results. These collections were analyzed with the same mitochondrial ND4 marker gene as in earlier studies [15],[16]. The same mosquitoes were assessed for VC and midgut infection and escape barriers using established protocols [8].

## Methods

### Mosquito collection

Mosquitoes were collected as larvae from the cities listed in Table 1. In each city multiple locations were visited (Figure 1) and at each location at least 3 separate breeding sites separated by at least 500 meters were sampled. These larvae were returned to the laboratory and emerged adults were individually examined to confirm that they were Ae aegypti. The 2003 collection was processed for analysis of vector competence and mtDNA markers; the 2004 collection only for mtDNA analyses. All experiments used F_1_–F_4_ mosquitoes to minimize effects of colonization and inbreeding.

**Table 1:** Locations, dates of collections, coordinate, and sample sizes of *Aedes aegypti* collections in Mexico.

State	City/Region	Date(s) m/y	Latitude	Longitude	n
Nuevo Leon	Monterrey North	7/96	N25°40′00.12″	W100°18′00.00″	57
	South	7/96	N25°28′00.12″	W100°10′01.20″	58
	West	7/96	N25°30′00.00″	W100°04′58.80″	58
	East	7/96	N25°40′59.88″	W100°22′01.20″	58
Tamaulipas	Ciudad Victoria	8/96	N23°40′00.12″	W099°15′00.00″	59
	Miguel Aleman	6/98	N26°23′30.00″	W099°03′39.00″	50
	Matamoros	7/96	N26°15′00.00″	W097°28′00.12″	59
	Nuevo Laredo	8/97	N27°30′00.00″	W099°28′00.12″	48
	Reynosa	7/97	N26°10′00.12″	W098°10′00.12″	59
	Tampico	8/96	N23°40′00.12″	W097°49′59.88″	59
Veracruz*	Panuco	08/03, 08/04	N22°03′12.47″	W098°11′11.78″	141
	Tantoyuca	08/03, 08/04	N21°20′30.33″	W098°13′39.88″	118
	Poza Rica	08/03, 08/04	N20°32′37.18″	W097°28′14.83″	105
	Martinez de la Torre	08/03, 08/04	N20°02′59.97″	W097°02′19.77″	102
	Acayucan	08/03, 08/04	N17°57′43.07″	W094°24′45.17″	138
	Alvarado	08/03, 08/04	N18°46′27.19″	W095°45′48.80″	116
	Coatzacoalcos	08/03, 08/04	N18°08′26.91″	W094°24′47.15″	120
	Cosoleacaque	08/03	N17°57′43.07″	W094°32′09.79″	63
	Minatitlan	9/96, 08/03, 08/04	N17°58′47.00″	W094°32′27.00″	161
	Moloacan	9/98	N17°59′09.00″	W094°20′46.00″	55
	Tuxpan	8/96	N21°10′00.12″	W097°25′00.12″	59
	Zempoala	08/03, 08/04	N19°26′41.60″	W096°24′23.31″	105
Tabasco	Villahermosa	9/98	N17°59′59.99″	W092°54′00.00″	58
Campeche	Campeche	8/98	N19°53′59.99″	W090°36′00.01″	53
	Cd. del Carmen	8/98	N18°35′59.99″	W091°47′59.99″	52
Yucatan	Merida	7/99	N20°57′00.01″	W089°38′23.99″	57
	North	7/99	N21°00′44.64″	W089°37′51.60″	49
	South	7/99	N20°57′06.84″	W089°38′26.88″	35
	Central	7/99	N20°57′58.68″	W089°39′57.24″	46
	East	7/99	N20°59′28.32″	W089°35′00.60″	53
	West	7/99	N20°58′39.00″	W089°39′28.80″	60
Quintana Roo	Cancun Central	6/99	N21°08′24.01″	W086°52′47.99″	32
	North	6/99	N21°09′03.61″	W086°52′38.97″	53
	Chetumal Central	6/99	N18°29′59.99″	W088°18′00.00″	38
	North	6/99	N18°30′29.31″	W088°17′49.97″	54
Total					2488

### Vector competence

The DENV2 strain used was dengue 2 JAM1409, which was isolated in 1983 in Jamaica and belongs to the American Asian genotype [18],[19]. Procedures for growing virus in 14 day cell culture, quantifying the virus and infecting mosquitoes with membrane feeders covered with sterile hog gut membranes are published [8]. A highly DENV2 susceptible *Aedes aegypti* colony called D2S3 [20] served as an internal control in each experimental feed to test for consistency in the titer and infectiousness of the DENV2 meal preparation. Undiluted virus titers ranged from 7.5–8.5 log_10_ infectious virus/ml, which resulted in infection of 100% of the D2S3 mosquitoes in each feeding experiment.

Fully engorged mosquitoes were removed from the feeding carton and held for 14-days at a constant 27°C and 80% relative humidity in an insectary with a 12-hour photoperiod. Mosquitoes were frozen at −70°C until processed. Heads and abdomen were assayed for infections by immunofluorescence assay (IFA) using a mouse derived primary monoclonal antibody directed against a flavivirus E gene epitope [21],[22]. DNA was then extracted from the thorax [23] for population genetic studies.

For IFA, detection of DENV antigen in head tissues revealed a disseminated infection; these mosquitoes were scored as head positive (H+). The H+ mosquitoes were considered to be vector competent (VC), because salivary glands become infected in disseminated DENV infections and the H+ mosquitoes are presumably capable of transmitting the virus [24]. If no viral antigen was detected in the head tissues, the mosquito was scored as head negative (H−) and vector incompetent (VIC). To determine the anatomic basis for VIC, the H− mosquitoes were then examined to determine if the midgut was infected. H− mosquitoes with no detectable antigen in the midgut were scored as having a midgut infection barrier (MIB). H− mosquitoes with detectable viral antigen in the midgut were scored as having a midgut escape barrier (MEB). Because mosquitoes with a MIB could not be phenotyped for a MEB, we also determined the overall head negative rate (H-R) = H−/N.

The 95% confidence interval around VC, MIB, MEB and H-R was calculated as the Wald interval:
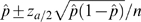
where 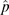 VC, MIB, MEB and H-R. Estimates are either adjusted by adding half of the squared Z-critical value (1.96) to the numerator and the entire squared critical value to the denominator before computing the interval [25].

### Population structure

Primers used to amplify the ND4 and all the polymerase chain reaction (PCR) and Single Strand Conformation Polymorphism (SSCP) conditions were reported earlier [15],[16]. The ND4 PCR products from mosquitoes containing each of the 9 haplotypes were sequenced at least once along both strands using an ABI sequencer (Davis Sequencing, Davis, California). Products from at least two mosquitoes representing each haplotype were sequenced. These 20 sequences were compared to sequences reported previously and assigned the same numeric labels [15],[16]. Phylogenetic relationships among haplotypes have been previously described [15],[16].

### Statistical analysis of mitochondrial haplotype frequencies

Variation in haplotype frequencies within and among collection sites and regions was examined using Molecular Analysis of Variance (AMOVA) [26]. Arlequin3 estimated pairwise Slatkin's “linearized F_ST_” [F_ST_/(1−F_ST_)] [27] among collections and computed the significance of the variance components associated with each level of genetic structure by a nonparametric permutation test with 100,000 pseudoreplicates [26]. A distance matrix containing linearized F_ST_ values was collapsed to construct a dendrogram using unweighted pair-group method with arithmetic averaging analysis [28] in the NEIGHBOR procedure in PHYLIP3.5C [29].

### Spatial analysis of vector competence

Inverse Distance Weighting interpolations are based on the assumption that the interpolating surface should be influenced most by the nearby points and less by the more distant points [30],[31]. The transformed VC values (arcsin√VC) were interpolated and the resulting surface was then back transformed. The maximum search area considered was 2.5° with no anisotropy (i.e. circular search area); the search was continued until five geographically most proximate collections (neighbors) were identified.

## Results

### Gene flow

The ND4 was amplified and surveyed for variation by SSCP analysis [32],[33] among 654 mosquitoes in 19 collections (Table 1). These were 10 collections obtained in 2003 and 9 obtained in 2004 (no mosquitoes were collected in Cosoleacaque). Nine different ND4 haplotypes were detected with SSCP. The ND4 gene was sequenced in 20 mosquitoes. All the sequenced haplotypes were compared to those previously reported (GenBank accession numbers AF334841–AF334865), and no novel haplotypes were detected. Accordingly all haplotypes in this study retain the same numerical designations as those in GenBank. As reported in previous studies [15],[16], sequences of mosquitoes with identical SSCP patterns were identical within each haplotype, and SSCP patterns differed among mosquitoes with one or a few nucleotide differences.

**Figure 2:**
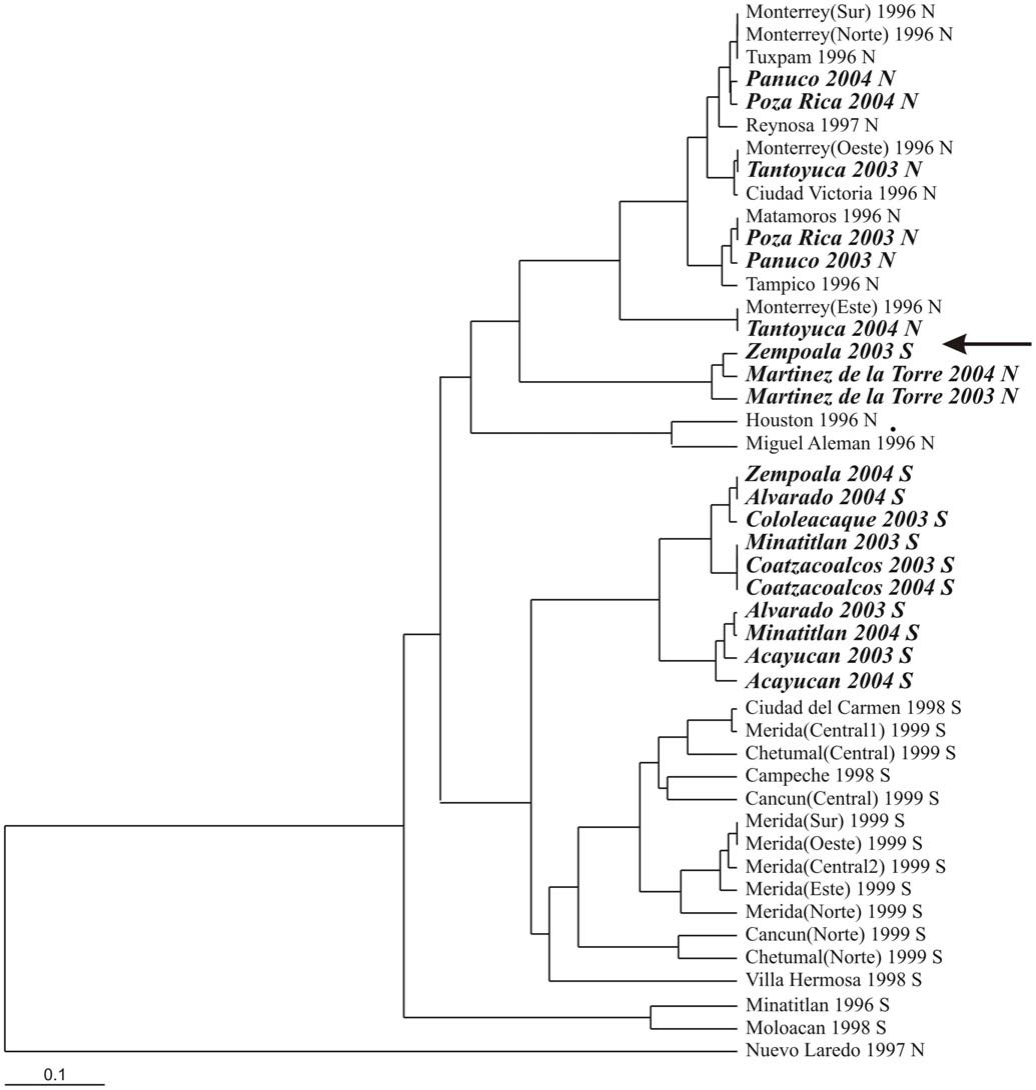
An UPGMA cluster analysis of pairwise linearized *F_ST_* values among 46 collections including 19 from the present study, 12 from previous studies north of Panuco in 1996–1997 [22] and 15 from south and east of Minatitlan in 1998–1999 [21].

**Figure 3:**
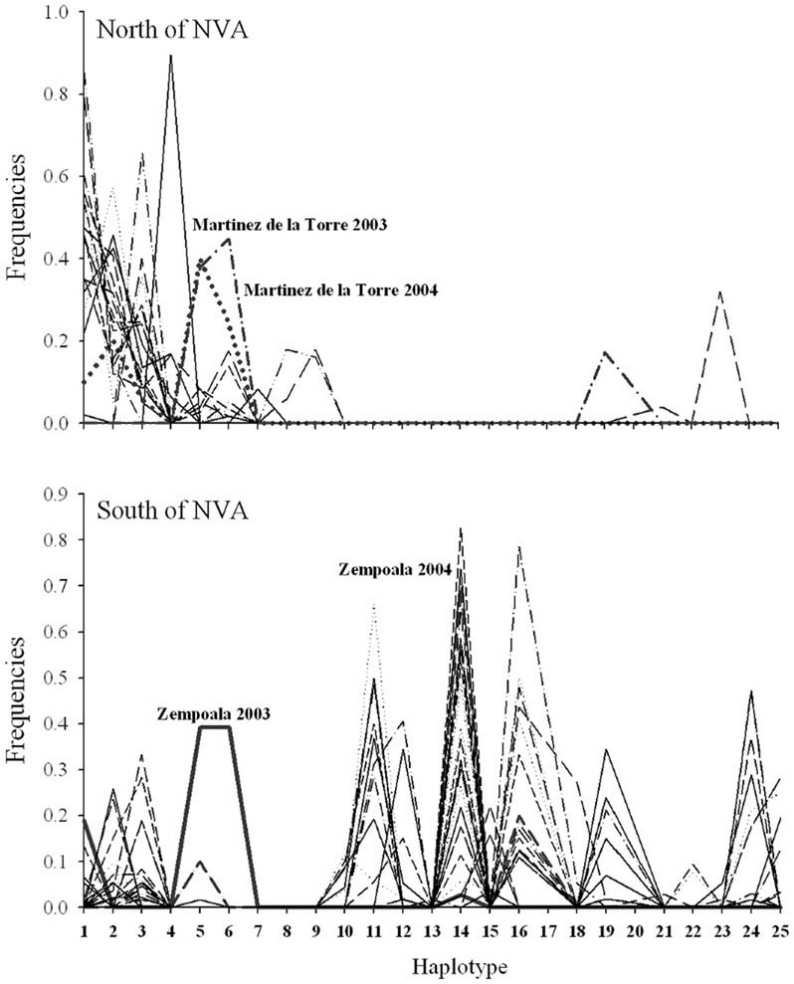
Relative frequencies of the 24 mitochondrial ND4 haplotypes in the 46 collections north (top) and south (bottom) of the Neovolcanic Axis. Haplotype number designations correspond to those in GenBank accessions AF334841–AF334865.

Figure 2 is a UPGMA cluster analysis of pairwise linearized *F_ST_* values [27] among 46 collections including 19 from the present study, 12 from previous studies north of Panuco in 1996–1997 [16] and 15 from south and east of Minatitlan in 1998–1999 [15]. With the single exception of Nuevo Laredo, all collections in northern Veracruz fall within a single cluster. The genetic distinctness of Nuevo Laredo *Ae. aegypti* was previously reported for both RAPD and mtDNA markers [16]. Northern collections cluster independently of collection year. Figure 3 indicates that most mosquitoes in northern Veracruz have haplotypes 1–9 and that haplotypes 10–18 are absent.

With the exceptions of Minatitlan 1996, Moloacan 1998, and Zempoala 2003, all collections in southern Veracruz fall into a cluster that is distinct from the northern cluster (Figure 2). Figure 3 indicates that most mosquitoes in southern Veracruz have haplotypes 10–18, 19 and 24. In 2003 Zempoala (Figure 1) mosquitoes (indicated with an arrow in Figure 2) clustered with the northern collections and were most similar to Martinez de la Torre (Figure 3), the collection site just north of Zempoala. But in 2004, Zempoala mosquitoes clustered with the southern collections. In contrast to northern mosquitoes, southern collections cluster according to collection year with 1998–1999 collections clustering independently of the 2003–2004 collections. Figure 2 indicates that the Minatitlan 1996 and Moloacan 1998 collections are similar to one another but genetically very distinct from Minatitlan 2003–2004. Moloacan and Minatitlan are in close geographic proximity to one another, yet they are also close to Cosoleacaque, Acayucan, and Coatzacoalcos.

### Nested analysis of haplotype frequencies

AMOVA [26] was used to compare haplotype frequencies 1) among all 46 collections in northern and southern Veracruz, 2) among the nineteen 2003 and 2004 collections in northern and southern Veracruz, and 3) in 2003 vs. 2004 collections (Table 2). When analyzing all 46 collections, a significant 16% of the variation in haplotype frequencies arose *between* collections in northern and southern Veracruz (Table 2) and an additional 20% arose *among* collections made either in northern or southern Veracruz. A similar pattern was detected when analyzing the 2003 and 2004 collections alone. However a greater percentage of the variation (24.5%) arose *between* collections in northern and southern Veracruz and, probably because we eliminated variation arising from the 1996–1998 collections, less variation (13%) arose among collections in northern and southern Veracruz. Whether collections were made in 2003 or 2004 made no difference. A negative and non-significant percentage of the variation arose between years.

**Table 2:** Molecular Analysis of Variance [26] of haplotype frequencies between the 46 collections north and south of the NVA, the nineteen 2003 and 2004 collections north and south of the NVA and between 2003 and 2004 collections.

Source of variation	d.f.	S.S.	Var. comp. (F)	%
**All 46 Collections**				
Collections N vs. S of the NVA	1	103.80	0.079 (F_NVA_ = 0.162)***	16.3
Among collections N or S of NVA	44	249.87	0.099 (F_Collections(NVA)_ = 0.243) ***	20.4
Within collections	2435	756.33	0.308 (F_(Mosquitoes(Collections)_ = 0.366)***	63.4
Total	2498	1109.91	0.487	
**19 collections from 2003-4**				
Collections N vs. S of the NVA	1	65.30	0.118 (F_NVA_ = 0.245)***	24.5
Among collections N or S of NVA	17	64.45	0.062 (F_Collections(NVA)_ = 0.172) ***	13.0
Within collections	1055	315.52	0.299(F_(Mosquitoes(Collections)_ = 0.375)***	62.5
Total	1073	445.26	0.479	
**2003 vs. 2004 collections**				
Between 2003 vs. 2004 collections	1	1.92	−0.011 (F_Year_ = −0.026)	−2.7
Among collections within years	17	127.82	0.128 (F_Collections(Year)_ = 0.300)***	30.8
Within collections	1055	315.52	0.299 (F_(Mosquitoes(Collections)_ = 0.282)***	71.9
Total	1073	445.26	0.416	

***: p-value ≤ 0.0001

### Analysis of vector competence

Table 3 provides the results of the vector competence studies for DENV2 of mosquitoes from the 2003 collections. In each site we report the proportion of mosquitoes with virus in the head tissues (H+) or not (H−), and for H− individuals the presence of virus in the midgut (M+) or not (M−). The VC (H+/N), VIC (H−/N), MIB (M−/N) and MEB (H−/M+) rates were calculated for each population (Table 3). The VC and VIC rates as well as the MIB rate for each of the 10 populations are presented in pie charts in Figure 1.

**Table 3:** Vector competence in the 2003 Veracruz Collections.

Collection	N	^1^M+	^2^M-	^3^H+	^4^H-	^5^VC	^6^95CI	^7^MIB	^6^95CI	^8^H-R	^6^95CI	^9^MEB	^6^95CI
Panuco	60	39	21	23	16	0.383	(0.264–0.503)	0.350	(0.232–0.468)	0.267	(0.156–0.377)	0.410	(0.263–0.558)
Tantoyuca	77	60	17	45	15	0.584	(0.477–0.692)	0.221	(0.128–0.313)	0.195	(0.106–0.284)	0.250	(0.142–0.358)
Poza Rica	47	41	6	35	6	0.745	(0.622–0.867)	0.128	(0.028–0.227)	0.128	(0.028–0.227)	0.146	(0.035–0.258)
Martinez*	72	61	11	37	24	0.514	(0.401–0.626)	0.153	(0.068–0.237)	0.333	(0.227–0.440)	0.393	(0.274–0.513)
	256	201	55	140	61	0.547	(0.486–0.607)	0.215	(0.165–0.265)	0.238	(0.186–0.290)	0.303	(0.240–0.367)
Zempoala	75	55	20	25	30	0.333	(0.229–0.438)	0.267	(0.168–0.366)	0.400	(0.292–0.508)	0.545	(0.418–0.673)
Alvarado	56	20	36	6	14	0.107	(0.021–0.193)	0.643	(0.521–0.765)	0.250	(0.138–0.362)	0.700	(0.511–0.890)
Cosoleacaque	63	33	30	8	25	0.127	(0.042–0.212)	0.476	(0.356–0.596)	0.397	(0.279–0.514)	0.758	(0.614–0.901)
Minatitlan	60	29	31	12	17	0.200	(0.099–0.301)	0.517	(0.394–0.639)	0.283	(0.171–0.395)	0.586	(0.417–0.755)
Acayucacan	73	35	38	8	27	0.110	(0.035–0.185)	0.521	(0.409–0.632)	0.370	(0.262–0.478)	0.771	(0.634–0.909)
Coatzasacoalcos	71	46	25	22	24	0.310	(0.204–0.415)	0.352	(0.243–0.461)	0.338	(0.230–0.446)	0.522	(0.383–0.660)
	398	218	180	81	137	0.204	(0.164–0.243)	0.452	(0.404–0.501)	0.344	(0.298–0.391)	0.628	(0.565–0.692)
P(Wilcoxon Test)						0.0095		0.0191		0.0667		0.0095	

* = Martinez de la Torre, ^1^ Midgut Positive, ^2^ Midgut Negative, ^3^ Head Positive, ^4^ Head Negative, ^5^ Vector Competence (VC) = H+/N, ^6^ Wald 95% Confidence interval (95CI), ^7^ Midgut Infection Barrier (MIB) = M−/N, ^8^ Head Negative Rate (H-R) = H−/N, ^9^ Midgut Escape Barrier (MEB) = H−/M+

In northern Veracruz, the VC rate ranged from 0.38–0.75 with an average of 0.55, while VC among mosquitoes in southern Veracruz ranged from 0.11–0.33 and averaged 0.20. These differences were significant (Wilcoxon tests for unpaired samples, *p* = 0.0095). This variation was attributable to the greater proportion of mosquitoes with both MIBs and MEBs in southern Veracruz. Mosquitoes with uninfected guts constituted 21.5% of collections in northern Veracruz while 45.2% of mosquitoes in southern Veracruz had a MIB. This 23.7% difference in MIB rate was significant (Wilcoxon tests for unpaired samples, *p* = 0.0191). MEB rate varied by 32.5% between northern MEB% = 30.3%) and southern (MEB% = 62.8%) collections ((Wilcoxon tests for unpaired samples, *p* = 0.0095).

The VC rates among the 10 Veracruz sites were interpolated by Inverse Distance Weighting (IDW) [30],[31] with (arcsin√VC)/100 using ArcInfo 9.1. The model derived by jackknifing over the 10 sites using the “leave-one-out” procedure had a R^2^ = 0.66 and a root mean square error of 9.6. The interpolated predicted values appear in colors from red (susceptible) to yellow (refractory) in Figure 1.

**Figure 4:**
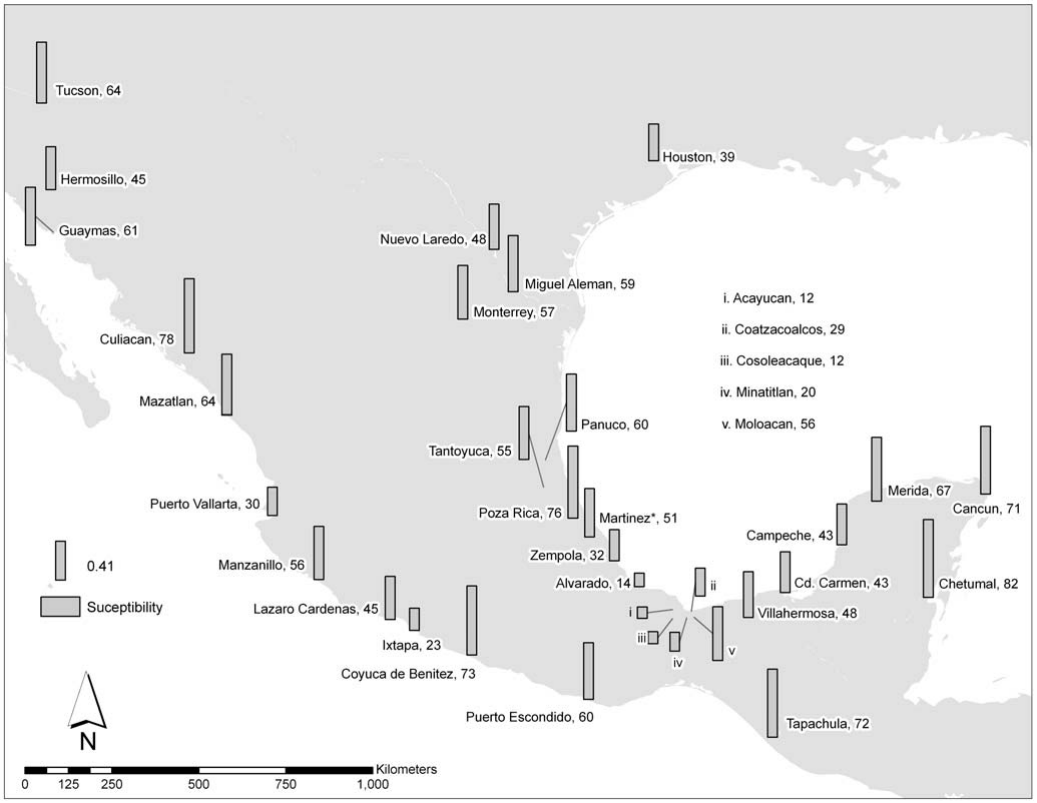
Map of Mexico and southern United States indicating the locations of the 34 collections of *Aedes aegypti*, 24 from a previous study [8] and 10 from the present study made over an 8 year period. The bars represent mosquito susceptibility to DENV2 (Jam1409). The number next to the city name is the mosquito susceptibility ((H+/N)×100).

Both the original measurements of VC (pie charts) and the predicted values from IDW interpolation suggest that VC declines precipitously south of the intersection of the Neovolcanic axis (NVA) with the Gulf of Mexico coast (Figure 1). The overall pattern in VC among 34 collections of *Ae. aegypti* made over an 8 year period throughout Mexico and two sites in the southern United States (Figure 4) demonstrates that the VC of mosquitoes from Alvarado, Acayucan, Coatzacoalcos and Cosoleacaque is among the lowest in Mexico.

## Discussion

Our results are consistent with an hypothesis that the intersection of the NVA with the Gulf of Mexico coast is the barrier to gene flow previously observed between *Ae. aegypti* collections north [16] and south on coastal plain along the Gulf of Mexico [15]. The Transverse Volcanic Belt of Mexico [34] divides the state of Veracruz into northern and southern Coastal Plains. This belt began to develop during the Oligocene and then later, during the Pliocene–Pleistocene, intense orogenic activity raised the Neovolcanic axis. The NVA extends from near the Pacific Coast east to the Gulf of Mexico and intersects the Atlantic coast in the state of Veracruz. The NVA favored a warm and dry climate in the south of Mexico, and promoted the establishment of tropical deciduous forests [35],[36]. Near the NVA, the onset of rainfall is earlier than in the semiarid highlands. Mexico's six highest mountains are part of the NVA, which constitutes the largest east west mountain range on the North American continent and have played an enormous role in vicariance and allopatric speciation events in a large number of plant and animal species [37]–[39]. We observed a local change in mitochondrial haplotype frequencies in Zempoala from a northern type of pattern (very similar to Martinez de la Torre) in 2003 to a more southern type of pattern (high frequency of haplotype 14) in 2004 (Figure 3). This suggests that local gene flow can occur across the NVA intersection. However, the overall pattern among collections made over an 8 year period (Figure 2) argues strongly that the narrow corridor between the NVA and the Atlantic Ocean restricts gene flow in the long term.

It isn't clear why the NVA acts as a barrier to gene flow in *Ae. aegypti*. We examined differences in climatic factors such as solar radiation, precipitation, and land use as potential barriers to gene flow in the Veracruz Coastal Plain, but there were no obvious consistent differences in these factors north and south of the NVA [40]. The NVA could serve as physical barrier to gene flow because the distribution of *Ae. aegypti* in Mexico is largely limited to elevations < 610 m (∼2,000 feet) above sea level [41]. However, elevations also exceed this limit where the NVA intersects the Pacific Ocean and mosquitoes from Tapachula north to Tucson Arizona appeared to represent a single panmictic population [15].

One major difference between the Pacific and Atlantic coasts of Mexico is the amount of movement of people and commerce. *Aedes aegypti* is generally considered to have low mobility via flight [42],[43], but is facilely moved about locally and globally through human transportation and commerce [14], [44]–[47]. In comparison with the Pacific Coast of Mexico, where there are major roads and railways (albeit not always on the edge of the coastal plain), the corridor between the Atlantic Ocean and the NVA contains only a single, two lane road that is used only for local travel. Most automobile and truck traffic between northern and southern Mexico goes through Mexico city [40]. The Pacific Coast also has robust maritime and cruise ship activity which may traffic *Ae. aegypti* along the coast. In contrast, there is little such activity between cities on the Gulf of Mexico, which could also limit gene flow [40]. Overall these considerations suggest that the principal barrier is human trafficking and commerce, but further investigations will be required to determine if this is true.

Unexpectedly the NVA is also associated with significantly different VC phenotypes. Figure 4 shows the overall pattern in vector competence of *Ae. aegypti* in Mexico and the southern United States and demonstrates that the low vector competence of mosquitoes from sites just south of the NVA is unusual. The reasons for this remain to be determined. The genetic mechanisms conditioning the differences in VC remain to be determined. Association mapping studies to determine if the early trypsin and late trypsin genes conditioned VC revealed no consistent associations between segregating sites in the genes and VC for DENV2 [48]. Importantly the DNA from each of the mosquitoes phenotyped for DENV2 VC has been archived and as new candidate genes for VC are identified, the potential role of the genes in VC can be rapidly tested using these materials. It is also important to note that these studies have been done with only one dengue virus serotype/genotype. It will be important to confirm these results with additional dengue serotypes and genotypes that are circulating in Mexico and Latin America [19].

The temporal stability of the VC patterns north and south of the NVA is of interest. VC for DENV2 appears to be a quantitative genetic trait with up to 60% of the variation in VC being associated with random, or uncontrolled environmental effects [9],[12]. QTL mapping of genome regions conditioning MIB and MEB have identified 8 different genome regions [11],[13],[14] three associated with a MEB, and five associated with an MIB and three of these mapping families originated from northeastern Mexico [11],[13],[14]. An ongoing reevaluation of VC in mosquitoes collected north and south of the NVA in 2005 indicates that the reduced VC south of the NVA is stable (S. Bernhardt, personal communication).

An interesting alternative hypothesis is that the patterns that we are detecting may have little to do with environmental and ecological factors and may instead represent the introduction of *Ae aegypti formosus* south of the NVA [46],[49]. The two subspecies are sympatric in Senegal [50] and other parts of West Africa, and could have been introduced independently and multiple times into the New World. We recently discovered chromosomal inversions in Senegalese *Ae. aegypti formosus* [51]. Such inversions might also act as barriers to gene flow if they condition prezygotic reproductive isolating mechanisms. If mating does occur then inversions might cause excess chromosome breakage following crossing over during meiosis in hybrids yielding aneuploid gametes. *Aedes aegypti aegypti and Ae aegypti formosus* differ dramatically in their vector competence for yellow fever virus [5],[7] and DENV [50]. Provocatively, the *Ae. aegypti* populations north and south of the NVA also differed significantly in VC for DENV2. However a focal distribution of *Ae. aegypti formosus* in southern Veracruz would not explain why no barriers to gene flow were detected between southern Veracruz collections and collections in the Yucatan Peninsula.

A major goal of our dengue research program in Mexico is to determine if mosquito VC is correlated with dengue incidence. If so, identification of genes that are biomarkers of VC could permit targeting of control efforts to areas at greatest risk for dengue epidemics. In this regard, it is intriguing that the *Ae. aegypti* south of the NVA exhibited low VC and some cities, for example, in Moloacan, the seroprevalence rate was zero [17]. This historical data, which is 20 years old, may not reflect current conditions in southern Veracruz. The epidemiology of dengue in Mexico has changed dramatically in the last two decades, and dengue is now hyperendemic in Coastal Plains of Mexico. A serosurvey for dengue antibodies conducted in Jatilpan, Veracruz, which is in the same region as Moloacan, revealed a seroprevalence rate of 80% [52]. However, in the Secretaria de Salud report there were cities/towns in southern Veracruz that had similar seroprevalence rates in 1986. Clearly this is a complex situation, and conducting prospective VC studies and seroprevalence surveys in cities and towns in this unique region. Such studies would provide important information on the importance of VC in dengue incidence.

## Supporting Information

Alternative Language Abstract S1 Translation of the Abstract into Spanish by Saul Lozano-Fuentes
(0.03 MB DOC)
