# Supplementary material for: The Neovolcanic Axis Is a Barrier to Gene Flow among Aedes aegypti Populations in Mexico That Differ in Vector Competence for Dengue 2 Virus
Source: PLoS Negl Trop Dis. 2009 Jun 30;3(6):e468. doi: 10.1371/journal.pntd.0000468 (PMC2697380; doi:10.1371/journal.pntd.0000468)
Supplement: Alternative Language Abstract S1 — Translation of the Abstract into Spanish by Saul Lozano-Fuentes (0.03 MB DOC) [file pntd.0000468.s001.doc]

## Alternative language abstract

**Antecedentes**

*Aedes aegypti* es el principal mosquito vector de los 4 serotipos del virus del Dengue (DENV, de sus siglas en ingles). Estudios previos de genetica de poblaciones y de competencia vectorial han demostrado en las poblaciones de *Ae. aegypti* en Mexico una clara estructuracion genetica y grandes diferencias en la habilidad de transmitir virus del dengue.

**Metodos/Principales descubrimientos**

Estudios de genetica de poblaciones han revelado que la interseccion del Eje Neovolcanico (NVA, de sus siglas en ingles) con la costa del Atlantico en el estado de Veracruz actua como una barrera distinguible de flujo genetico entre las poblaciones de *Ae. aegypti* al norte y al sur del NVA. Las poblaciones de mosquitos al norte y sur del NVA tambien son diferentes en su competencia vectorial (VC). La tasa promedio de VC en *Ae. aegyti* al norte del NVA fue de 0.55; en contraste el promedio de las poblaciones al sur de del NVA fue de 0.20. Gran parte de la variacion puede ser atribuida a una barrera de infeccion en el intestino medio; 21.5% de los mosquitos al norte del NVA y 45.2% de los mosquitoes al sur del NVA no desarrollaron infecciones del intestino medio.

**Conclusiones**

Barreras al flujo genetico en poblaciones de vectores pueden tambien impactar la frecuencia de genes que condicionan rasgos continuos y epidemiologicamente relevantes como lo es la competencia vectorial. Estudios subsecuentes son requeridos para determinar el porque el NVA es una barrera de flujo genetico asi como establecer si las diferencias observadas en competencia vectorial al norte y sur del NVA son estables y epidemiologicamente significativas.
